# Effects of acupuncture combined with medication on patients with COVID-19 complicated with bipolar disorder: A protocol of systematic review and meta-analysis

**DOI:** 10.1097/MD.0000000000031474

**Published:** 2022-11-11

**Authors:** Wenjing Huang, Luwen Zhu, Minmin Wu, Lili Teng, Mei Zhang, Wenjing Song

**Affiliations:** a Heilongjiang Administration of Traditional Chinese Medicine, Harbin, China; b The Second Affiliated Hospital of Heilongjiang University of Chinese Medicine, Harbin, China; c Heilongjiang University of Chinese Medicine, Harbin, China.

**Keywords:** acupuncture, acupuncture combined with medication, bipolar disorder, COVID-19, meta-analysis, protocol

## Abstract

There is growing evidence that coronavirus disease 2019 (COVID-19) can trigger acute episodes of mood disorders or psychotic symptoms. Reports on the treatment of COVID-19-related bipolar disorder (BD) are limited. Our study aimed to investigate the potential for new or recurrent BD due to COVID-19. We qualitatively evaluate clinical treatments (acupuncture combined with medication) and any potential pathophysiological links between infection and BD.

**Methods:**

We searched Embase, PubMed, Cochrane Library, Web of Science and MEDLINE (via Web of Science), Scopus, Chinese Biomedical Literature Database (CBM), Chinese National Knowledge Infrastructure Database, and the Wanfang Database from December 1, 2019, to September 15, 2022, to identify all articles on acupuncture combined with drugs used to treat COVID-19 complicated with bipolar disorders. Two researchers will screen the articles and extract the relevant information.

**Results:**

The results will provide a systematic overview of the current evidence on the use of acupuncture combined with drug therapy to treat COVID-19 complicated with bipolar disorder.

**Conclusion:**

The conclusions of this study will help clarify the effects of acupuncture combined with drug therapy on patients with COVID-19-related BD.

## 1. Introduction

According to the World Health Organization, since the first case of coronavirus disease 2019 (COVID-19) was reported in the Wuhan region of China in December 2019, the COVID-19 pandemic has caused more than 680 million infections worldwide.^[1,2]^ The pandemic has sparked fear of contracting the virus, and has challenged people with social precautions; as a result, it has had an unprecedented impact on people’s lives. COVID-19 is caused by infection with severe acute respiratory syndrome coronavirus 2 (SARS-CoV-2) and is usually accompanied by symptoms such as fever, cough, and fatigue.^[3]^ Although the main clinical manifestations of COVID-19 infection include respiratory symptoms, other manifestations are increasingly reported.^[4]^ Early reports indicate residual effects of SARS-CoV-2 infection, such as fatigue, dyspnea, chest pain, cognitive impairment, arthralgia, and reduced quality of life.^[5–7]^ The impact of the COVID-19 pandemic on mental health has also been a concern, with reports of various neuropsychiatric manifestations in patients with COVID-19 around the world.^[8]^ The occurrence of neuropsychiatric symptoms in patients with COVID-19 has been extensively explored over the past 2 years.^[9,10]^ In susceptible individuals, neuroinflammation and neuroinvasion caused by Sars-CoV-2 may trigger a cascade of events, causing neurotransmitter and neuroendocrine imbalances, which in turn affect the control of excitatory and inhibitory circuits, and leading to bipolar disorder (BD) or psychotic symptoms.^[11]^

The COVID-19 pandemic has caused unprecedented disruption around the world, which may disproportionately affect people with mental illness, especially those with BD. BD is a common, life-long disabling psychiatric disorder, characterized by the presence of 1 or more recurrent manic (BD-I), hypomanic, and depressive (BD-II) episodes, which may alternate rapidly. The estimated lifetime prevalence of any type of BD ranges from 0.5% to 5%.^[12,13]^ Because these patients are more affected by altered biological and social rhythms, the diagnosis of COVID-19 and subsequent hospitalization, psychological distress, and neuropsychiatric manifestations of the disease can lead to BD recurrence.^[14]^ BD may be triggered by biological, psychological, and social factors, or potentially by neuroinvasion of the virus.^[15]^ COVID-19 infection leads to the secretion of cytokines and the activation of the kynurenine pathway of tryptophan metabolism, and these chemicals enhance disorders of limbic circuits, which may be associated with psychiatric disorders such as psychosis, BD, depression, and suicide.^[16]^ Studies shown that some people with no previous history of mental illness develop specific symptoms after being infected with SARS-CoV-2,^[17]^ including acute onset of mood disorders or psychotic symptoms.^[18]^ Mood changes, such as anxiety and insomnia, are common after SARS-Cov-2 infection, however, the incidence of post-COVID-19 bipolar disorder may be underestimated. BD is also a well-known risk factor for suicide and is accompanied by lifelong mood disorders, and COVID-19 infection can lead to recurrence or development of BD.^[14,17]^ Therefore, BD should be properly treated and managed after COVID-19.

At present, the treatment for patients with BD mainly includes drug therapy as a main treatment with non-drug therapy as a supplement.^[19]^ In recent years, the treatment status of patients with BD has improved with the continuous development and application of drugs. Evidence-based treatment of BD includes the use of lithium, anticonvulsants, and atypical antipsychotics.^[20]^ Studies have shows that most patients do not achieve symptom relief and functional recovery with these drugs alone or in combination with existing treatments.^[21]^ This, along with side effects (such as weight gain), high recurrence, and high cost, has led to high treatment discontinuation and conversion rates of patients with BD.^[22]^ Based on the fact that during the outbreak of the COVID-19 pandemic, it has a greater impact on patients with BD and newly diagnosed patients with BD caused by COVID-19, the current BD treatment remains difficult and the prognosis is still poor, and western medicine treatment inevitably has the disadvantages of large side effects, easy recurrence, and high cost.^[23]^ Therefore, non-drug therapy and supplementary alternative drug therapy as adjuvant therapy are particularly important. In Eastern cultures, acupuncture has been used for thousands of years to treat a range of ailments, including mental disorders. A study evaluating the safety and reliability of adjuvant acupuncture to treat BD have reported that all patients improved symptoms after treatment, although the results were not significant.^[24]^ A small Australian study found significantly greater improvement in depressive symptoms with active laser acupuncture than with sham laser acupuncture.^[25]^ A recent study conducted in China showed good effects of the treatment of first-episode bipolar disorder with nape acupuncture combined with ear acupuncture, and there was no statistical difference between the follow-up and the 4-weeks treatment, indicating that acupuncture can produce long-term effects on BD patients.^[26]^ In addition, some studies have shown that acupuncture and moxibustion can reduce drug toxicity and adverse drug reactions^[27,28]^ and can relieve other pulmonary and non-pulmonary symptoms after COVID-19.^[29]^ However, the effect of acupuncture combined with drug therapy on COVID-19-related BD is currently unclear. Although acupuncture has achieved positive results in previous studies, these studies are few, and stronger evidence is needed to prove this. Therefore, we will conduct a systematic review and meta-analysis to evaluate the efficacy and safety of acupuncture combined with medication in the treatment of COVID-19-related BD.

## 2. Methods

### 2.1. Protocol registration

In accordance with the guidelines, this systematic review protocol was registered with the International Prospective Register of Systematic Reviews (PROSPERO) on September 21, 2022 (registration number CRD42022361801), which will be conducted in accordance with preferred Reporting Items for Systematic Review and Meta-Analysis Protocols (PRISMA-P) 2015 statement guidelines.^[30]^

### 2.2. Inclusion criteria for study selection

#### 2.2.1. Types of studies.

This review will include randomized controlled trials of acupuncture combined with medication in patients with COVID-19 with BD. There is no language and publications limitation. Non- randomized controlled trials, observational study, reviews, experimental studies, clinical case reports, and animal research will be excluded.

#### 2.2.2. Participants.

Patients with COVID-19 with BD will be included, regardless of age, gender, educational status, or racial restrictions. The diagnosis of COVID-19 will be based on international diagnostic criteria^[31]^ and the diagnosis of BD will be based on *Diagnostic and Statistical Manual of Mental Disorders, Fourth Edition* (*DSM-IV*) criteria for bipolar disorder (type I or II).

#### 2.2.3. Types of interventions.

All types of acupuncture therapies combined with medication will be included in this study. Studies must include at least 1 of the following comparators: acupuncture therapies combined with medication versus pure acupuncture therapy; acupuncture therapies combined with medication versus pure medication treatment; and acupuncture therapies combined with medication versus sham acupuncture combined with medicine or acupuncture combined with placebo. Uncertain interventions will be excluded.

#### 2.2.4. Outcomes.

Primary outcomes

1. The current severity of the disorder will be measured with the Clinical Global Impression for Bipolar Disorder (CGI-BP).^[32]^
2. Symptoms will be assessed with the Brief Symptom Inventory (BSI), which is the short version of the SCL-90.^[33]^
3. Depressive symptoms will be measured with Hamilton Depression Rating Scale (HAM-D)^[34]^ and Manic symptoms will be assessed with the Bech-Rafaelsen Mania Scale (MAS).^[35]^

Secondary outcomes

Quality of life and social functioning will be measured by the Personal and Social Functioning Scale^[36]^ and the Brief Quality of Life in Bipolar Disorder Questionnaire. (QoL.BD).^[37]^

### 2.3. Search strategy

A literature search will be performed using 9 electronic medical databases: Embase, PubMed, Cochrane Library, Web of Science and MEDLINE (via Web of Science), Scopus, Chinese Biomedical Literature Database (CBM), Chinese National Knowledge Infrastructure Database (CNKI), and the Wanfang Database, from December 1, 2019, to September 15, 2022, to identify all articles on COVID-19 with bipolar disorders treated with acupuncture combined with drugs. In addition, to ensure that no relevant studies were missed, we traced references to the full text that had been identified. We use a search strategy that combines subject terms with free words. The search strategy for PubMed is presented in Table 1.

**Table 1:** PubMed search strategy.

Search	Query
#1	“Acupuncture Therapy”[MeSH Terms] OR “acupuncture treatment”[Title/Abstract] OR “acupuncture treatments”[Title/Abstract] OR “treatment acupuncture”[Title/Abstract] OR “therapy acupuncture”[Title/Abstract] OR “pharmacoacupuncture treatment”[Title/Abstract] OR ((“therapeutics”[MeSH Terms] OR “therapeutics”[Title/Abstract] OR “Treatments”[Title/Abstract] OR “Therapy”[MeSH Subheading] OR “Therapy”[Title/Abstract] OR “Treatment”[Title/Abstract] OR “treatment s”[Title/Abstract]) AND “Pharmacoacupuncture”[Title/Abstract]) OR “pharmacoacupuncture therapy”[Title/Abstract] OR ((“therapeutics”[MeSH Terms] OR “therapeutics”[Title/Abstract] OR “therapies”[Title/Abstract] OR “Therapy”[MeSH Subheading] OR “Therapy”[Title/Abstract] OR “therapy s”[Title/Abstract] OR “therapys”[Title/Abstract]) AND “Pharmacoacupuncture”[Title/Abstract]) OR “Electroacupuncture”[MeSH Terms]
#2	“Drug Therapy”[MeSH Terms] OR “therapy drug”[Title/Abstract] OR “drug therapies”[Title/Abstract] OR “therapies drug”[Title/Abstract] OR “Pharmacotherapy”[Title/Abstract] OR “Pharmacotherapies”[Title/Abstract]
#3	#1 AND #2
#4	“COVID-19”[MeSH Terms] OR “COVID-19”[Title/Abstract] OR “sars cov 2 infection”[Title/Abstract] OR “infection sars cov 2”[Title/Abstract] OR “sars cov 2 infection”[Title/Abstract] OR “sars cov 2 infections”[Title/Abstract] OR “2019 novel coronavirus disease”[Title/Abstract] OR “2019 novel coronavirus infection”[Title/Abstract] OR “2019 ncov disease”[Title/Abstract] OR “2019 ncov disease”[Title/Abstract] OR “2019 ncov diseases”[Title/Abstract] OR “disease 2019 ncov”[Title/Abstract] OR “covid 19 virus infection”[Title/Abstract] OR “covid 19 virus infection”[Title/Abstract] OR “covid 19 virus infections”[Title/Abstract] OR ((“infect”[Title/Abstract] OR “infectability”[Title/Abstract] OR “infectable”[Title/Abstract] OR “infectant”[Title/Abstract] OR “infectants”[Title/Abstract] OR “infected”[Title/Abstract] OR “infecteds”[Title/Abstract] OR “infectibility”[Title/Abstract] OR “infectible”[Title/Abstract] OR “infecting”[Title/Abstract] OR “infection s”[Title/Abstract] OR “Infections”[MeSH Terms] OR “Infections”[Title/Abstract] OR “Infection”[Title/Abstract] OR “infective”[Title/Abstract] OR “infectiveness”[Title/Abstract] OR “infectives”[Title/Abstract] OR “infectivities”[Title/Abstract] OR “infects”[Title/Abstract] OR “pathogenicity”[MeSH Subheading] OR “pathogenicity”[Title/Abstract] OR “infectivity”[Title/Abstract]) AND “covid 19 virus”[Title/Abstract]) OR “virus infection covid 19”[Title/Abstract] OR “coronavirus disease 2019”[Title/Abstract] OR “disease 2019 coronavirus”[Title/Abstract] OR “coronavirus disease 19”[Title/Abstract] OR “coronavirus disease 19”[Title/Abstract] OR “severe acute respiratory syndrome coronavirus 2 infection”[Title/Abstract] OR “sars coronavirus 2 infection”[Title/Abstract] OR “covid 19 virus disease”[Title/Abstract] OR “covid 19 virus disease”[Title/Abstract] OR ((“COVID-19”[Title/Abstract] OR “COVID-19”[MeSH Terms] OR “covid 19 vaccines”[Title/Abstract] OR “covid 19 vaccines”[MeSH Terms] OR “covid 19 serotherapy”[Title/Abstract] OR “covid 19 serotherapy”[Supplementary Concept] OR “covid 19 nucleic acid testing”[Title/Abstract] OR “covid 19 nucleic acid testing”[MeSH Terms] OR “covid 19 serological testing”[Title/Abstract] OR “covid 19 serological testing”[MeSH Terms] OR “covid 19 testing”[Title/Abstract] OR “covid 19 testing”[MeSH Terms] OR “SARS-CoV-2”[Title/Abstract] OR “SARS-CoV-2”[MeSH Terms] OR “severe acute respiratory syndrome coronavirus 2”[Title/Abstract] OR “nCoV”[Title/Abstract] OR “2019-nCoV”[Title/Abstract] OR ((“Coronavirus”[MeSH Terms] OR “Coronavirus”[Title/Abstract] OR “CoV”[Title/Abstract]) AND 2019/11/01:3000/12/31[Date - Publication])) AND “virus diseases”[Title/Abstract]) OR “disease covid 19 virus”[Title/Abstract] OR “virus disease covid 19”[Title/Abstract] OR “2019 ncov infection”[Title/Abstract] OR “2019 ncov infection”[Title/Abstract] OR “2019 ncov infections”[Title/Abstract] OR “infection 2019 ncov”[Title/Abstract] OR “COVID19”[Title/Abstract] OR “covid 19 pandemic”[Title/Abstract] OR “covid 19 pandemic”[Title/Abstract] OR “pandemic covid 19”[Title/Abstract] OR “covid 19 pandemics”[Title/Abstract]
#5	“Bipolar Disorder”[MeSH Terms] OR “bipolar disorders”[Title/Abstract] OR “disorder bipolar”[Title/Abstract] OR “psychosis manic depressive”[Title/Abstract] OR “psychosis manic depressive”[Title/Abstract] OR “psychoses manic depressive”[Title/Abstract] OR “psychoses manic depressive”[Title/Abstract] OR “manic depressive psychosis”[Title/Abstract] OR “manic depressive psychosis”[Title/Abstract] OR “bipolar mood disorder”[Title/Abstract] OR “bipolar mood disorders”[Title/Abstract] OR “disorder bipolar mood”[Title/Abstract] OR “mood disorder bipolar”[Title/Abstract] OR “affective psychosis bipolar”[Title/Abstract] OR “bipolar affective psychosis”[Title/Abstract] OR “psychoses bipolar affective”[Title/Abstract] OR “psychosis bipolar affective”[Title/Abstract] OR “depression bipolar”[Title/Abstract] OR “bipolar depression”[Title/Abstract] OR “manic depression”[Title/Abstract] OR “depression manic”[Title/Abstract] OR “depressions manic”[Title/Abstract] OR “manic disorder”[Title/Abstract] OR “disorder manic”[Title/Abstract] OR “manic disorders”[Title/Abstract]
#6	#4 AND #5
#7	#3 AND #6

### 2.4. Data collection and analysis

#### 2.4.1. Selection of studies.

**Figure 1.**
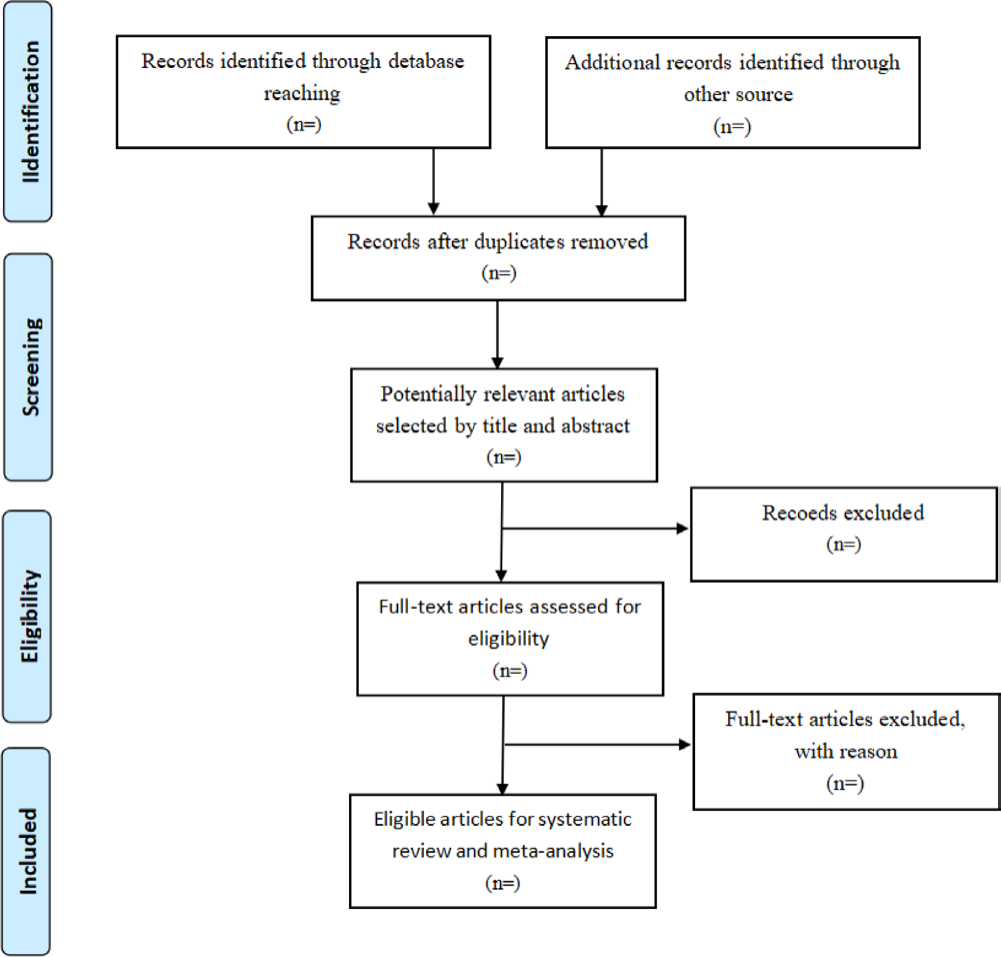
The study selection process.

Two reviewers (W-JH and L-WZ) will independently review and screen the studies according to the inclusion and exclusion criteria of the review. EndNote V.X9 (Clarivate Analytics, Pennsylvania) will be used to manage the search results from above-mentioned databases. Two reviewers (W-JH and L-WZ) will independently identify articles by title and abstract according to inclusion criteria, and ineligible or duplicate studies will be removed. Next, 2 reviewers will perform full-text screening of the remaining literature, and the 3^rd^ reviewer (W-JS) will solve any disagreement between the first 2 reviewers. The process of screening selection is shown in Figure 1.

#### 2.4.2. Data collection.

Two reviewers (M-MW and W-JS) will independently screen the literature and extract data from the included studies. The data extraction form will include basic information: first author, year of publication, country, study design (study type, sample size, age and sex of participant, and duration), interventions (type of acupuncture, acupoint selection, number of sessions, frequency of treatment, duration of each session, name of drug, dose of drug, and frequency of use), control (name, dose, frequency, and duration of treatment), outcome measures (type and number of adverse events in each group), primary outcomes, and secondary outcomes. The completed data extraction forms will be cross-checked by the 2 reviewers (M-MW and W-JS). Any discrepancies will be resolved by the third reviewer (L-WZ).

#### 2.4.3. Assessment of risk of bias.

Two reviewers (W-JH and L-LT) will assess the risk of bias for each included study by the *Cochrane Handbook for Systematic Reviews of Interventions* (The Cochrane Collaboration, 2011)^[38]^: selection bias: random sequence generation and allocation concealment; performance bias: blinding of participants and study personnel to the condition; detection bias: blinding of outcome assessment; attrition bias: incomplete outcome data; reporting bias: selective outcome reporting of results for BD; and other bias. The risk of bias in each aspect will be assessed, and the results will be categorized into 3 grades: low risk, unclear risk, and high risk. The assessment results will be cross-checked by the 2 reviewers and the disagreements will be resolved by the third reviewer (W-JS).

#### 2.4.4. Assessment of quality of evidence.

The Grading of Recommendations Assessment, Development, and Evaluation system will be used to judge the overall quality of evidence supporting outcomes in this work. The quality of evidence will be defined as high, moderate, low, or very low.

### 2.5. Data synthesis and analysis

#### 2.5.1. Data synthesis and assessment of heterogeneity.

The Stata 15.1 software (Stata Statistical Software: College Station, TX: Stata Corp LP) will be used to meta-analyze the extracted data. The mean difference (MD) with 95% CI will be used for continuous variables, and standardized MD and 95% CI will be used for continuous variables if the units are different. The Q test and I^2^ statistic were used to evaluate the heterogeneity of merged studies. I^2^ values of approximately 25%, 50%, and 75% indicate low, moderate, and substantial heterogeneity, respectively. When significant heterogeneity exists, a random-effects model will be used; otherwise, a fixed-effect model will be used.

#### 2.5.2. Subgroup analysis.

If the included studies show obvious clinical heterogeneity, subgroup analysis will be conducted according to clinical characteristics. In this study, we will conduct subgroup analysis according to the type of acupuncture, type of medicine, treatment time, severity of BD at baseline, and primary or recurrent BD.

#### 2.5.3. Sensitivity analysis.

We will use sensitivity analysis to verify that the results are robust. Methodological quality, sample size, and the effect of missing data will be included. Therefore, the impact of low-quality studies on the overall results will be evaluated.

#### 2.5.4. Meta-regression analysis.

If the heterogeneity cannot be resolved after subgroup analysis, we will perform a meta-regression analysis on the characteristics of the article, such as country, publication year, included population characteristics, etc. Meta-regression will be conducted when there are at least 10 studies in a meta-analysis.^[38]^

#### 2.5.5. Assessment of reporting biases.

Reporting bias, including publication bias, time lag bias, duplicate publication bias, and outcome reporting bias will be assessed by funnel plot, if the number of included studies exceeds 10.^[38]^

#### 2.5.6. Grading the quality of evidence.

We will use the Grading of Recommendations Assessment, Development, and Evaluation Reliability Study system to assess the quality of the obtained evidence.

#### 2.5.7. Dealing with missing data.

We will attempt to contact the corresponding author for more detailed information regarding missing or unclear data. If this fails, we will analyze the available data.

### 2.6. Ethics and dissemination

Due to the nature of systematic review and meta-analysis, ethical approval is not required. The results of this study will be disseminated through a peer-reviewed journal.

## 3. Discussion

The spread of the COVID-19 pandemic has brought serious health consequences to every medical field, including mental health. Infection with SARS-CoV-2 can affect the mental health of psychiatric patients, aggravate previous mental diseases, and lead to new mental diseases. Some research results have raised questions about the possibility that SarscoV-2 infection may trigger manic/hypomanic episodes (BD-I), which are becoming more frequent in the context of COVID-19.^[15]^ Awareness should be raised even if the patient has no previous psychiatric history. As 1 of the main treatment methods of BD, medicine can achieve some therapeutic effect, but drug treatment of BD still has a high recurrence rate, poor prognosis, and other problems.^[39]^ Acupuncture, a non-drug treatment, can be used as a safe auxiliary, and can reduce drug toxicity. The combination of drug therapy and non-drug therapy can reduce the suicide rate of patients with BD. Therefore, in the future, studies related to non-drug therapy for BD should be strengthened, and more attention should be paid to the effectiveness and safety of drug therapy combined with non-drug therapy in order to inform the treatment of BD and improve the prognosis.

## Author contributions

Wenjing Huang and Luwen Zhu share first authorship.

**Conceptualization:** Luwen Zhu.

**Data curation:** Wenjing Song, Minmin Wu.

**Formal analysis:** Wenjing Huang, Wenjing Song.

**Investigation:** Minmin Wu, Lili Teng.

**Methodology:** Wenjing Huang, Minmin Wu, Lili Teng, Mei Zhang.

**Project administration:** Luwen Zhu.

**Resources:** Minmin Wu.

**Software:** Wenjing Huang.

**Supervision:** Luwen Zhu.

**Visualization:** Lili Teng, Mei Zhang.

**Writing – original draft:** Wenjing Huang, Wenjing Song.

**Writing – review & editing:** Luwen Zhu, Minmin Wu.
